# Oropharyngeal carcinomas induce circulating monocytes to express a TAM-like pro-tumor expression profile that suppresses T-cell proliferation

**DOI:** 10.3389/fimmu.2025.1539780

**Published:** 2025-03-19

**Authors:** Christopher J. Papayannakos, Mohd Israr, James A. DeVoti, Fung Lam, Arnon Arazi, Douglas K. Frank, Dev P. Kamdar, Lucio M. Pereira, Nagashree Seetharamu, Bettie M. Steinberg, Vincent R. Bonagura

**Affiliations:** ^1^ Northwell, New Hyde Park, NY, United States; ^2^ Northwell, New Hyde Park, NY and Feinstein Institutes for Medical Research, Northwell Health, Manhasset, NY, United States; ^3^ Northwell, New Hyde Park, NY and Cohen Children’s Medical Center, Queens, NY, United States; ^4^ Northwell, New Hyde Park, NY and Department of Otolaryngology, Jong Island Jewish Medical Center, New Hyde Park, NY, United States; ^5^ Northwell, New Hyde Park, NY and Department of Medicine, Zucker School of Medicine at Hofstra/Northwell, Hempstead, NY, United States; ^6^ Northwell, New Hyde Park, NY and Department of Molecular Medicine, Zucker School of Medicine at Hofstra/Northwell, Hempstead, NY, United States

**Keywords:** oropharyngeal cancer, tumor associate macrophages (TAM), T-cell suppression, spheroid, monocytes, single Cell RNA sequencing

## Abstract

**Introduction:**

Tumor-associated macrophages (TAMs) recruited from circulating monocytes drive tumor-growth and establish an immunosuppressive tumor microenvironment (TME). Initial events in transition from resting monocytes to TAMs are poorly understood. Here, we report that monocytes from oropharyngeal cancer (OPC) patients and control monocytes treated with OPC-conditioned media (CM) express a repertoire of pro-tumor mediators that is characteristic of TAMs.

**Methods:**

Monocytes were stimulated with OPC cell line CM, analyzed by single-cell RNAseq. Results of select genes were confirmed by qPCR with monocytes and analyzed in OPC tumors vs. clinically normal tissue. OPC spheroids containing control monocytes and T-cells were established, TAM phenotype characterized by flow analysis and qPCR, and T-cell proliferation assessed by flow.

**Results:**

OPC-conditioned media induced multiple pro-tumor genes including *CXCL1, CXCL5, CXCL8, SPP1, IL1B, GPNMB*, and *FABP5*. Patient monocytes had higher baseline levels or achieved higher levels after stimulation than control monocytes. A subset of patient monocytes had high baseline levels of *CXCL9/-10/-11* expression that resisted downregulation in response to stimulation, a potential sign of a more favorable TME. *CXCL9/-10/-11* expression in OPC tumor biopsies compared to clinically normal tissue correlated with patient outcome. Spheroid TAMs derived from control monocytes maintained the pro-tumor repertoire seen with monocytes stimulated by tumor line conditioned media. These TAMs suppress T-cell proliferation. Inhibition of COX-2 or IL1 signaling during differentiation into TAMs partially blocked the suppression of T-cell proliferation.

**Conclusion:**

Targeting the early transition of monocytes into pro-tumor TAMs could be used to develop new therapies for OPC.

## Introduction

Monocytes that differentiate into tumor associated macrophages (TAMs) are often the most abundant immunocytes in the tumor microenvironment (TME), and they play a major role in tumor development and progression (1–3). As tumors arise, local inflammation initiates recruitment of circulating monocytes which infiltrate tissues via tumor-derived chemokines, including CCL2 and CCL20 (4–7). Monocytes are then ‘educated’ by established tumors cells, leading to monocyte differentiation into TAMs (8). In many tumors, only a small fraction of TAMs arises from tissue-resident macrophages while the majority are derived from infiltrating monocytes (9). Phenotypically, TAMs express a repertoire of both M1-like (CD14, CD16, CD80, CD86, HLA-DR, CD11b) and M2-like (CD163, CD204, CD206, CD209) macrophage markers (10). As TAMs accumulate in the TME they influence inflammation, promote tumor cell migration, and polarize T-cells away from anti-tumor function (11, 12). There are gaps in understanding the initial impact of the tumor on circulating monocytes prior to their extravasation into the tumor and their differentiation into TAMs (13, 14).

TAMs are essential in shaping the TME through their secreted cytokines and chemokines that recruit additional immunocyte populations into the tumors. Tumor-derived CXCL5 recruits myeloid derived suppressor cells (MDSC), major contributors to the development of an immunosuppressive TME (15). Similarly, TAM derived CXCL7 and CXCL1 recruit and activate neutrophils, and in addition they promote angiogenesis, induce epithelial to mesenchymal transitioning, and promote tumor cell migration (16–20). TAMs also affect the recruitment and subsequent phenotype of infiltrating T-cells, recruiting regulatory T-cells (T-regs) and inducing T-cell exhaustion (21).

Oropharyngeal squamous cell carcinoma (OPC) is predominantly induced by human papillomaviruses (22). OPC is a significant global clinical problem with cases expected to increase by 50% over the next 20 years (23). Patient prognosis is correlated with TAM abundance in both HPV ^+ve^ and HPV^-ve^ OPCs, and TAMs are an important target for developing immunotherapies for this and other cancers (24, 25). We recently reported that prostaglandin E2 (PGE_2_) is increased in the plasma from OPC patients, and that these patient’s monocytes show elevated levels of cyclooxygenase-2 (COX-2) expression, the enzyme that leads to PGE_2_ synthesis (26). PGE_2_ in the TME helps drive pathogenic inflammatory TAM phenotypes, stabilizes myeloid derived suppressor cells, increases TAM accumulation in tumors, and supports angiogenesis across cancer types (27–29). We also showed that conditioned media (CM) from cultured primary OPC biopsies and established OPC cell lines induce COX-2 expression in peripheral blood monocytes from healthy controls but more robustly in patient-derived monocytes, with IL-1α in the CM contributing to these effects (26). IL-1α can have both pro-tumor and anti-tumor effects, and its role in solid tumor development is controversial (30). This communication builds on our earlier studies, expanding our understanding of the process of early monocyte education by the tumor (or products thereof) as they begin to differentiate into TAMs, in spheroid models of OPC tumors.

## Methods

Peripheral blood samples from OPC patients and healthy controls with no evidence of OPC, and biopsies of OPC tumors and clinically normal adjacent tissues from patients, were obtained after written consent, approved by Northwell Health’s IRB.

### Cell culture

SCC-25 (HPV16^-ve^) and SCC-154 (HPV16^+ve^) cell lines were purchased from the American Type Culture Collection (ATCC) (Manassas, VA) and maintained in E-medium DMEM/Ham’s F-12 (Gibco, Grand Island, NY) at a 3:1 ratio, supplemented with 10% Fetal Clone II (Hyclone, Logan, UT), 0.4 μg/mL hydrocortisone, 5 μg/mL transferrin, 2 nM 3,3-5-triodo-L-thyronine, 5ng/mL EGF, 5μg/mL insulin, (Sigma, Saint Louis, MO), and 100 units/mL penicillin and 100 μg/mL streptomycin (Gibco, Grand Island, NY). Cultures were maintained at 37°C in 5% CO_2_ and sub-cultured when they reached 70–80% confluence.

### Conditioned media generation

SCC-25 and SCC-154 cell lines were seeded in E-medium at 5.0 x10^4^ cells/mL on 60 mm culture plates. When approximately 50% confluent, cells were washed once with PBS and media replaced with E-media supplemented with 2% Fetal Clone II. Media was conditioned for 48 hours, collected, and centrifuged for 10 minutes at 700 RCF followed by 20 minutes at 3300 RCF at 4C. Clarified CM was then passed through a 0.22μm Steriflip filter (Millipore, Burlington, MA) before storage at -80C in 1mL aliquots.

### Monocyte isolation

20 mL of heparinized blood was diluted to 30 mL with RPMI 1640 culture medium (Life Technologies Limited, Paisley, U.K.) supplemented with 100 units/mL penicillin, 100 μg/mL streptomycin and 2mM L-glutamine, layered over Ficoll-Paque Plus (GE Healthcare, Uppsala, Sweden), and then centrifuged for 20 min at room temp at 500 RCF. The PBMC layer was collected and washed twice with RPMI 1640. Monocytes were isolated by negative selection using the Pan Monocyte Isolation Kit (Miltenyi Biotec, Bergish Gladbach, Germany), as per the manufacturer’s instructions. Only monocytes with a purity greater than 90% were used in subsequent experiments.

### Monocyte stimulation

Monocytes were plated in 250uL fresh E-medium +2% FCII at 10^6^ cells/mL and incubated with an equal volume of SCC-25 CM, SCC-154 CM, or unconditioned 2% E-medium for 18 hours. Monocyte RNA was isolated using RNeasy isolation kit (Qiagen, Aarhus, Denmark). For single cell sequencing, monocytes were also stimulated with 200 pg/mL recombinant human IL1α (R&D, Minneapolis, MN).

### Single cell RNA sequencing

Following stimulation, viability was assessed, cells were partitioned (Chromium Comptroller, 10X Genomics, Pleasanton, CA), and single cell libraries were prepared according to manufacturer’s instructions. Paired end sequencing was performed on both libraries. Raw data was demultiplexed, QC’d and aligned to hg38 followed by analysis using the Seurat package in R. Downstream analysis was performed using a custom single-cell Seurat pipeline. Briefly, low quality cells were filtered out based on proportion of reads mapping to mitochondrial genes, as were contaminating lymphocytes. Libraries were then normalized, data was scaled, and top 2000 variable features were identified and used for Principal Component Analysis. Libraries were batch corrected using Harmony. Nearest neighbors were found for graph-based clustering and projected using UMAP. Clustering was performed using a final resolution of 0.75, which maximized the number of clusters while minimizing cluster marker redundancy. Finally, differential expression was performed using FindAllMarkers function in Seurat.

### Reference mapping

Individual cells in the dataset were matched with a correlation reference table representing transcriptomes of previously described monocyte/DC subtypes (31). Indices were used to assess the most similar reference cluster, and the correlation score with that cluster. Each cell was colored according to its most similar reference cluster and projected as UMAP.

### Tissue RNA isolation

Genomic DNA and total RNA were isolated simultaneously from matched sets of tumor and clinically normal tonsil/base of tongue biopsies from each OPC patient using the AllPrep DNA/RNA Mini Kit (Qiagen, Hilden, Germany) as per manufacturer’s instructions. Expression of specific transcripts was analyzed by qRT-PCR as described below.

### qRT-PCR

Total monocyte or TAM RNA was isolated using the RNeasy Mini Kit (Qiagen, Hilden, Germany) as per manufacturer’s instructions and digested with the RNase-Free DNase-1 Set (Qiagen, Hilden, Germany) to remove contaminating genomic DNA. Reverse transcription and amplification were performed with the iTaq Universal Probes One-step Kit (BIO-RAD, Hercules, CA) as per manufacturer’s instructions. TaqMan gene expression assays comprised of intron-spanning primers and gene specific probes were purchased from Applied Bio-systems. A GAPDH-specific assay, Hs99999905_m1, was used to measure mRNA expression of this housekeeping gene in individual samples for comparison to target mRNA expression in total RNA samples using the ddCt method. All samples were amplified on Roche LightCycler480 II.

### Spheroid and TAM generation

10^4^ SCC-25 or SCC-154 cells, or normal foreskin keratinocytes, were cultured per well in ultra-low attachment 96-well U-bottom plates (S-Bio, NH, USA) in E-medium for 5 days. Then 10^4^ freshly isolated human monocytes were added to the spheroids in each well. Monocytes were not pre-selected for co-culturing based on phenotype; total monocytes were used after pan-monocyte isolation. The co-cultures were incubated for 7 additional days to generate spheroids with TAMs. Media was changed every 2-3 days.

### TAM phenotyping

*S*pheroids containing macrophages were collected, dissociated by incubation with StemPro™ Accutase™ Cell Dissociation Reagent (Gibco, Grand Island, NY, USA) at 37 °C and carefully re-suspended by pipetting up and down every 10 minutes. Cells were surface stained for TAM markers using antibodies to CD45 APC, CD14 APC-H7, CD11b PE, CD86 PE-Cy7, HLA-DR FITC, CD163 PerCP-Cy 5.5 and CD206 BV421 (BD Biosciences). Flow cytometry was performed on a BD FACS Canto II (BD Biosciences) and analyzed using FCS Express (*De Novo* Software). Macrophages were determined by positive stain of CD45 and further gated using forward and side scatter to exclude debris and nonviable cells.

### TAM sorting

TAMs were dissociated from spheroids using StemPro Accutase as stated above. The cell suspensions were washed with MACS buffer (Miltenyi Biotec, 130-091-376 & 130-091-222), surface stained with anti-CD45, washed, and resuspended in MACS buffer prior to sorting using FACS Aria II (BD Biosciences).

### T-cell proliferation assay

T-cells were isolated from PBMC by negative selection using the Pan T- Cell Isolation Kit (Miltenyi Biotec, Bergisch Gladbach, Germany) according to the manufacturer´s instructions. T-cells were washed with PBS and labeled with 5μM carboxyfluorescein succinimidyl ester (CFSE) (Invitrogen, C34554) following manufacturer’s instructions. 5x10^4^ labeled T-cells per well were then added to tumor-cell spheroids, tumor-cell spheroids containing polarized TAMs, or foreskin spheroids and foreskin spheroids containing macrophages. As proliferation controls, T-cells were cultured in E-media only. To activate T-cells, anti-CD3 and anti-CD28 activation beads (Invitrogen, #11131D) were added at a ratio of 1 bead to 10 T-cells. For inhibitor experiments, 25 ng/ml Anakinra (IL-1α inhibitor; Swedish Orphan Biovitrum, Stockholm, Sweden) and 1µM NS-398 (COX-2 Inhibitor; Sigma-Aldrich, Saint Louis, MO, USA) were added at the time of monocyte co-culture. Inhibitors were replaced when media was changed. Spheroids were dissociated using StemPro Accutase, washed with stain buffer, and T-cell proliferation assay was determined on day 6 using FACS Canto II (BD Biosciences).

### Statistics

Statistical tests were chosen based on normality and variance assessments of each data set using Shapiro and Levene’s tests in R. Analysis of monocyte subtype distribution and CXCL9/10/11/SPP1 expression changes were not found to be normally distributed and nonparametric tests were utilized. T-cell suppression assay data required a mixed effects model with *post-hoc* tests to account for monocyte/T-cell donor and spheroid type variables. A p-value <0.05 was considered significant.

### Data availability

Single cell data set is available in the NCBI Gene Expression Omnibus via Accession number GSE281098.

## Results

### OPC patients have elevated levels of non-classical monocytes compared to controls

**Figure 1:**
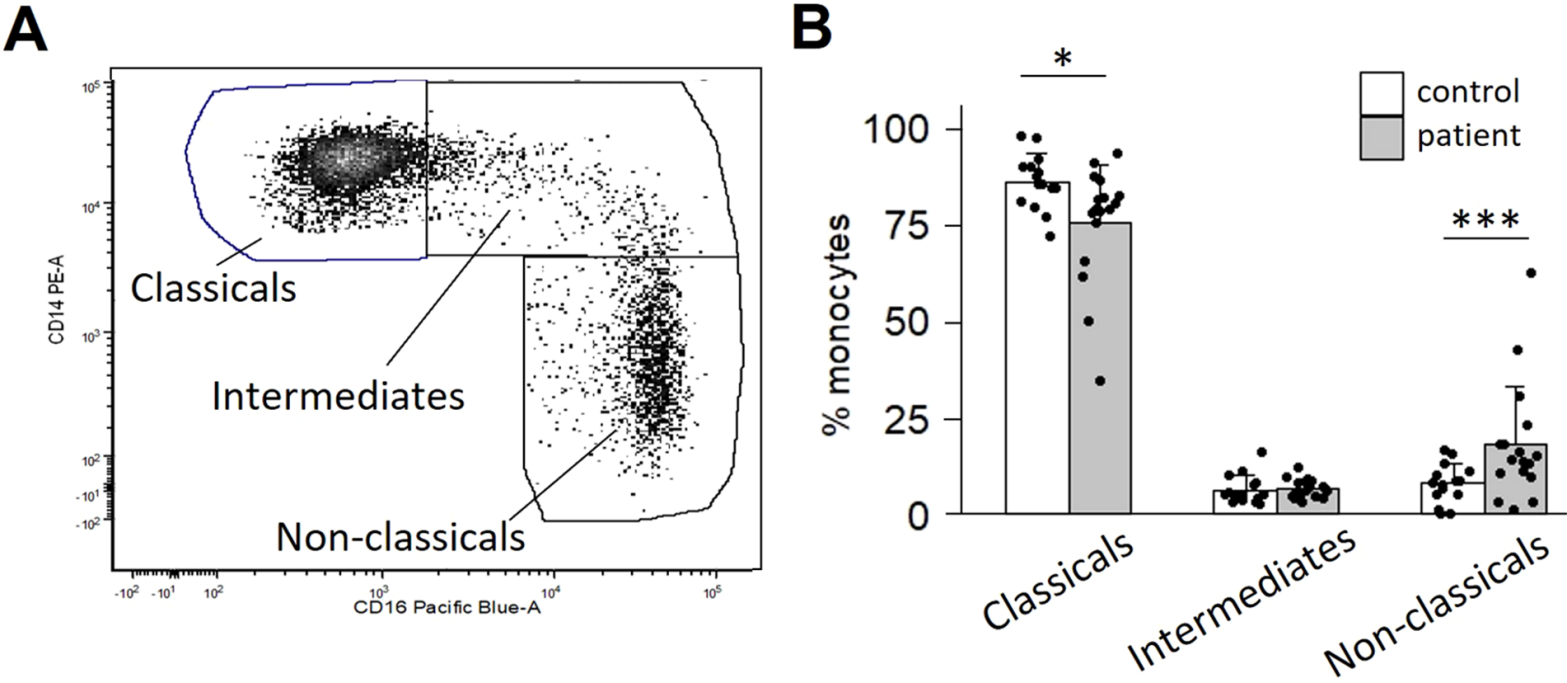
OPC patients display a skewed ratio of monocyte subtypes. Monocytes were isolated from peripheral blood by negative selection and stained for CD14 and CD16. **(A)** Scatter plot of a representative patient’s CD14/CD16 monocyte distribution. **(B)** Quantification of subtype distribution comparing 17 patients vs. 15 controls. *p<0.05; ***p<0.001, determined by Mann-Whitney analysis.

Monocytes have traditionally been classified into three subtypes based on CD14/CD16 surface expression. We first asked whether the proportion of these three subtypes differed between OPC patients compared to healthy controls. A typical flow analysis of monocytes from an OPC patient with the classical (CD14^++^/CD16^-^), intermediate (CD14^++^/CD16^+^) and non-classical (CD14^lo^/CD16^+^) subtypes is shown in **Figure 1A**. The majority of monocytes are classical. However, patients showed a reduced fraction of classical monocytes (p=0.018) and an elevated fraction of non-classical monocytes (p=0.008), with no difference in intermediate monocytes (p=0.427) (**Figure 1B**). Non-classical monocytes have a much longer half-life than classical monocytes (32), and thus are more likely to be influenced by soluble factors released by a tumor.

### OPC cell line-conditioned media induces monocyte expression of many genes, including pro-tumor cytokines and chemokines

**Figure 2:**
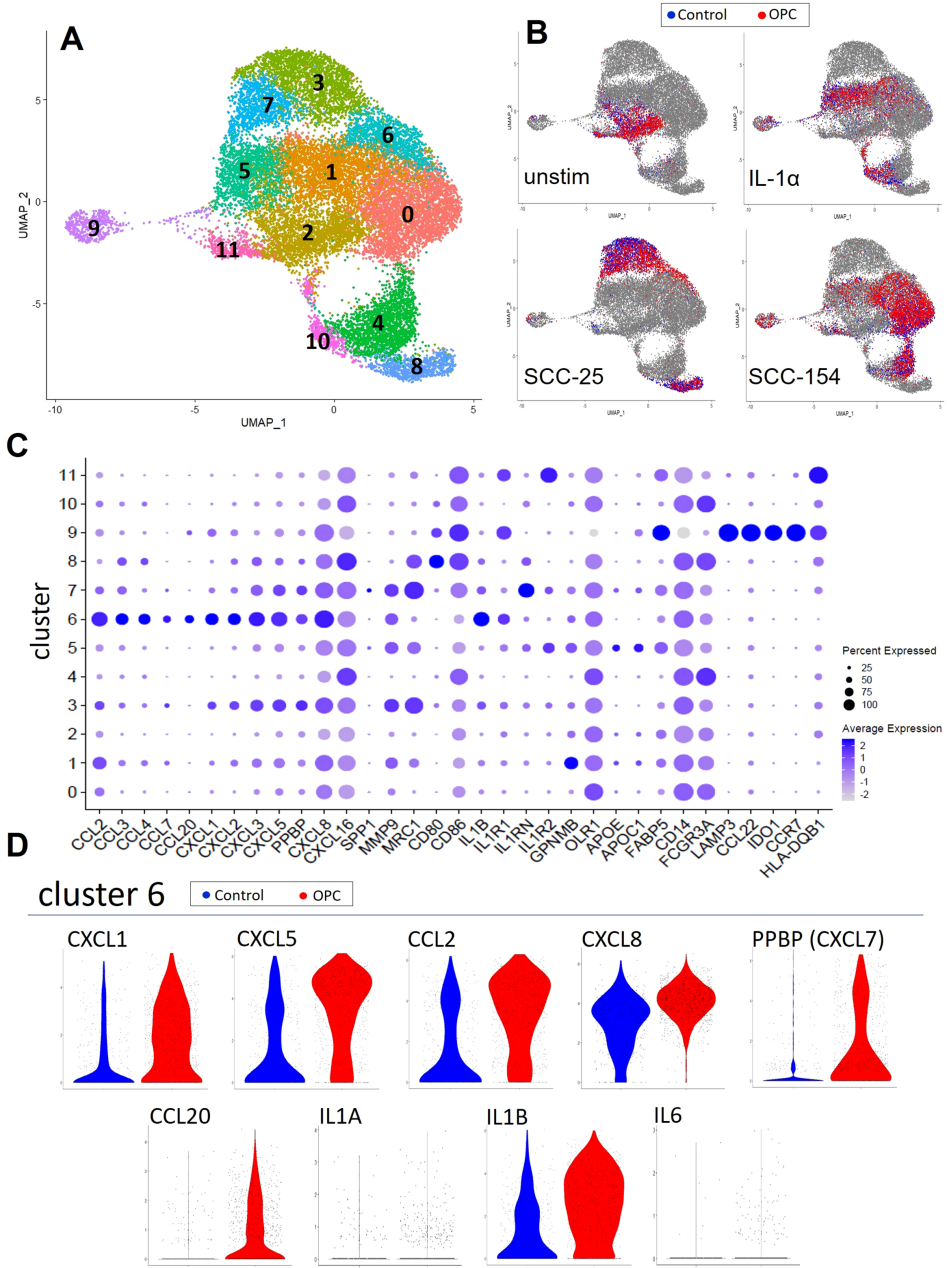
Single-cell RNA-sequencing captures monocyte response diversity to OPC cell-line conditioned media. Monocytes from a healthy donor and an OPC patient were enriched by negative selection from PBMC and stimulated for 18 hours with conditioned media from SCC-154 or SCC-25 cell lines, IL1α (100 pg/mL), or left in fresh E-media +2% FCII followed by single cell RNA sequencing. **(A)** UMAP of all 10195 (control) and 9153 (OPC) monocytes post-stimulation showing 12 unique cell clusters. **(B)** Stratified UMAPs with patient (red) and control (blue) monocytes from each stimulus shown over all other cells. **(C)** Single cell expression data showing genes relevant to TAM development. Features plotted were derived from the FindAllMarkers function (average log2 fold change threshold = 0.25) in Seurat, post-clustering. **(D)** Differential expression violin plots showing elevated cluster 6 markers in our OPC patient library.

We next investigated the early monocyte responses to OPC cell line soluble factors using scRNA-seq. *W*e compared monocyte transcriptomes of an OPC patient and a control after incubation with SCC-25-CM, SCC-154-CM, recombinant IL-1α, or unconditioned E-media. After sequencing and quality control, 9153 patient and 10195 control monocytes were analyzed. Clustering was performed at a resolution allowing for non-redundant clusters that aligned with treatment groups. Twelve clusters were visualized as a UMAP plot (**Figure 2A**). Reference mapping to monocyte/DC subsets previously described in Villani et al. (31) showed most of our cells classified as Mono1, transcriptionally akin to classical monocytes, followed by a minor group classified as Mono2, akin to non-classical monocytes, with trace numbers of Mono3 cells (**Supplementary Figure S1A**). Additionally, small numbers of dendritic-like cells correlated with conventional dendritic cells (DC1, DC2, DC3) and the less-well known DC4 and DC5 subsets. Mono1 and Mono2 subsets overlap well with CD14 and CD16 transcript patterns in the monocytes, validating our clustering strategy (**Supplementary Figure S1B**).

The relationship between the clusters and the type of stimulation is shown in **Figure 2B**. Unstimulated monocytes from both the patient and control were primarily localized in cluster 2 and were largely superimposable. SCC-154-CM and IL-1α stimulations resulted in partially overlapping responses in several clusters, while clusters 3, 7 and 8 arose nearly exclusively from SCC-25-CM stimulation.

**Figure 2C** shows the level of expression of over 40 immune and pro-tumor genes by cells within each cluster, including a multitude of inflammatory cytokines/chemokines (*IL1B, CXCL1, CXCL5, PPBP (CXCL7), CCL2, CXCL8*), extracellular matrix components and modulators (*FN1, COL23A1, VCAN, TGFBI, MMP9*), and genes indicating altered lipid metabolism (*APOE, APOC1, GPNMB, FABP5*). Cluster 9 has a dendritic cell-like pattern with high expression of *LAMP3, IDO1, CCL22, CCR7* and several class II MHC genes.

Cluster 6 represents a subset of monocytes defined by high expression of inflammatory/pro-tumor cytokines and chemokines including *IL1B, CXCL8, CCL3, CCL4, CXCL3, CCL20, CXCL2, PPBP (CXCL7)*, and *CCL7*. This cluster is composed of cells from all three treatment conditions. The ratio of patient to control cells in cluster 6 was 3:1, suggesting that this subset of the patient monocytes might have been more responsive to stimulation. Moreover, differential expression violin plots (**Figure 2D**) showed that the patient monocytes in cluster 6 expressed higher levels of many of these cytokines/chemokines. Most of the cluster 6 cells from these two individuals did not express measurable levels of *IL6* or *IL1A* despite our previously observing their induction in stimulated control monocytes.

### Treatment of monocytes from a large cohort of patients and controls with SCC-154 and SCC-25 CM confirmed increased expression of pro-tumor cytokines/chemokines, with higher levels for several in patients’ monocytes

**Figure 3:**
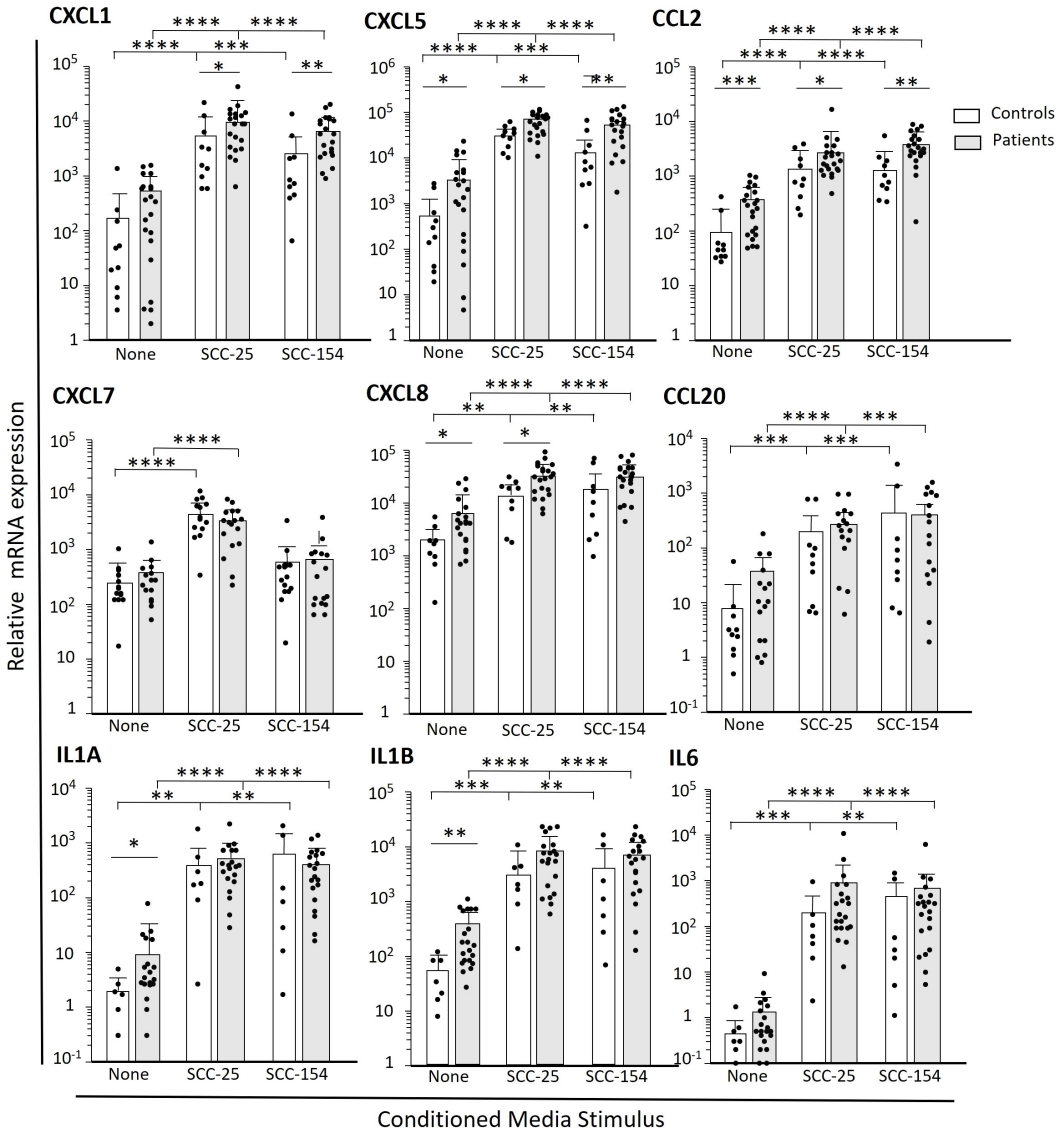
Select cytokines and chemokines are more robustly induced in patient-derived monocytes after short term challenge with OPC-cell line CM. Monocytes were enriched from PBMC from OPC patients and healthy controls by negative selection. Cells were stimulated with cell culture media conditioned by SCC-154, SCC-25 or unconditioned E-media as a control for 18 hours. Transcript levels were measured by qPCR and expressed relative to GAPDH. Results were analyzed using a Student’s t-test. *p<0.05; **p<0.01, ***p<0.001, ****p<0.0001.

The RNA sequencing shown in **Figure 2** provided us with a broad account of genes induced by OPC cell line CM, and intriguing differences between the patient and control within the cluster 6 subpopulation of monocytes. We then compared expression levels of select cytokines/chemokines by q-RT-PCR in CM-stimulated monocytes from a large cohort of patients and controls (**Figure 3**). Levels of the transcripts for the various immune mediators differed markedly, both at baseline and after stimulation, with *IL1A* and *IL6* especially low at baseline. There was marked stimulation of expression of cytokines/chemokines by both control and patient monocytes after treatment with conditioned media, with most increasing by 100 to 1000-fold (except for CXCL7 stimulation with SCC-154). Many of the transcripts were significantly elevated in the patients’ monocytes compared to controls at baseline (*CXCL5, CXCL8, CCL2, CCL20, IL1A*, and *IL1B*) and several, but not all, maintained that difference after stimulation. *CXCL1* transcript levels did not differ significantly in unstimulated monocytes but were higher in patients’ monocytes than control monocytes after stimulation.

### Monocytes from a subset of patients express high levels of *CXCL9/10/11* and are resistant to suppression by OPC-CM. These cells are absent in healthy controls

**Figure 4:**
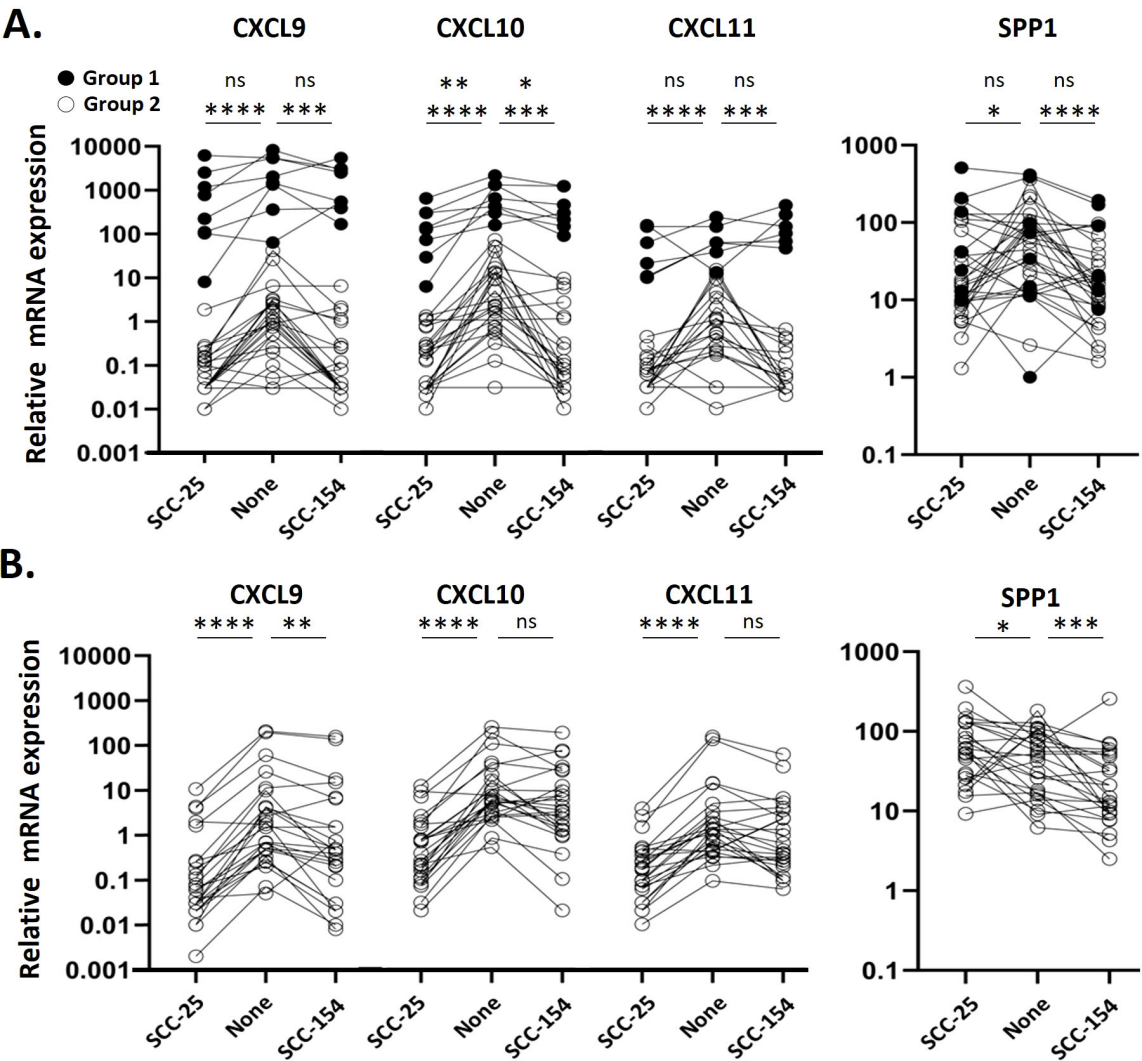
Monocytes from a subset of patients express high levels of CXCL9/10/11 and are resistant to suppression by CM. Monocytes were enriched from PBMC from OPC patients and healthy controls by negative selection prior to stimulating them with SCC154, SCC25 or unconditioned E-media for 18 hours. Target transcripts were measured by qPCR and expressed relative to GAPDH. **(A)** Expression levels in stimulated OPC patient monocytes. Filled points represent Group 1 patients, defined as a monocyte mRNA expression ratio of CXCL9:SPP1 >3, a subpopulation of patients not evident in controls. Open points represent Group 2 patients, with a CXCL9:SPP1 ratio < 3. **(B)** Expression levels in stimulated control monocytes. Results were analyzed using a Kruskal-Wallis test. *p<0.05; **p<0.01, ***p<0.001, ****p<0.0001, ns, not significant.

Although we did not observe *CXCL9, -10, or -11* expression in our single cell data set, we considered changes in their expression in our larger cohort due to the recently reported importance of these genes as markers of prognosis (33). Monocytes from patients clearly were in two groups (**Figure 4A**), which we defined as ≥ 3-fold greater ratio of *CXCL9* vs *SPP1* expression (Group 1) or < 3-fold ratio (Group 2). Monocytes from patients in Group 1 had very high levels of *CXCL9, -10* and –11 transcripts after incubation in unconditioned media. Expression of CXCL9 and CXCL11 by Group 1 monocytes were not suppressed by treatment with either CM, and CXCL10 levels were only modestly reduced. In contrast, monocytes from most patients (Group 2) had relatively low levels of expression of *CXCL9/10/11* at baseline, each of which were significantly reduced with CM treatment by either SCC-25 or SCC-154 CM (p<0.001). *SPP1* is thought to be expressed in opposition to *CXCL9*, since others reported that they were not normally expressed in the same cell. We saw no difference in *SPP1* levels between Group 1 and Group 2 patients in unstimulated monocytes. However, the mean level of *SPP1* expression went down in Group 2 monocytes after stimulation with SCC25 CM (p<0.05) and with SCC154 CM (p<0.0001) but did not change significantly in Group 1’s monocytes, suggesting that the Group 1 monocytes might be resistant to CM downregulation of expression of these chemokines. Monocytes from control donors, except for one, were comparable to Group 2 patients’ monocytes, with low levels of *CXCL9/10/11* transcripts that decreased further with CM treatment (p<0.001 for both treatments) (**Figure 4B**). Control monocytes had varied *SPP1* responses to stimulation.

### SCC-154 and SCC-25 spheroids induce monocytes to differentiate into TAMs

**Figure 5:**
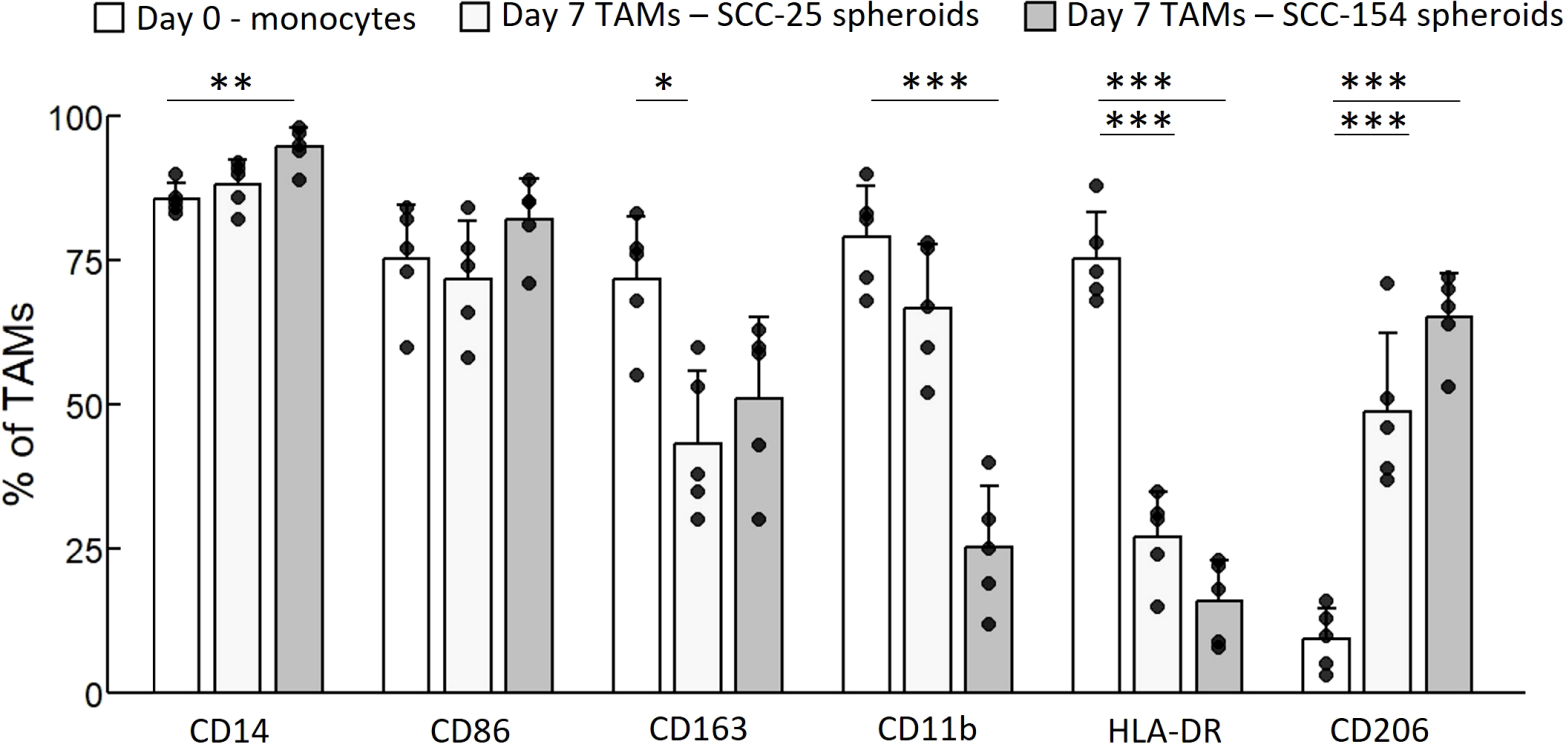
SCC-154 and SCC-25 spheroids modulate TAM marker expression in primary monocytes. Primary monocytes were isolated from healthy control blood by negative selection. 10^4^ monocytes/well were co-cultured with either 5-day old SCC-25 or SCC-154 spheroids for 7 additional days. Spheroids were dissociated with Accutase, all cells stained with conjugated antibodies and analyzed by flow cytometry. CD45-positive cells were assessed for expression of TAM-associated markers. Day-0 monocytes were similarly stained for comparison. Data is expressed as a percent of positive staining cells over all CD45+ cells. Results were analyzed using ANOVA for each marker. *p<0.05; **p<0.01, ***p<0.001.

To study the transition from monocytes into TAMs in OPC, and their impact on T-cells, we used a spheroid model generated with SCC-25 and SCC-154 cells, inserting control monocytes into the spheroids to generate TAMs, and inserting T-cells after the TAMs were established. As a control, we generated spheroids with human foreskin keratinocytes (HFKs) and inserted monocytes and T-cells. We first characterized the macrophage surface markers of TAMs derived from tumor spheroids and macrophages derived from HFK spheroids by flow cytometry and compared them to day-0 monocyte expression (**Figure 5**). The marker CD14 was slightly upregulated by SCC-154 spheroids, unsurprising as CD14 expression increases during the differentiation of monocytes into macrophages. The M1 marker CD86 did not change from day 0 monocytes while the M1 marker HLA-DR was markedly reduced. The M2 marker CD163 was robustly downregulated (p = 0.01) on SCC-25 TAMs, while slightly down on SCC-154 TAMs (p = 0.06). In contrast, the M2 marker CD206 was upregulated on TAMs in both spheroids. Finally, CD11b, a critical regulator of pro-inflammatory immune responses expressed on both M1 and M2 macrophages, was markedly reduced on SCC-154 TAMs. Together, phenotypic assessment of our *in vitro* TAMs demonstrates the mix of M1- and M2- markers previously described for TAMs isolated from tumors (34–36).

### Spheroid-induced TAMs maintain the cytokine/chemokine profile induced by CM stimulation of monocytes

**Figure 6:**
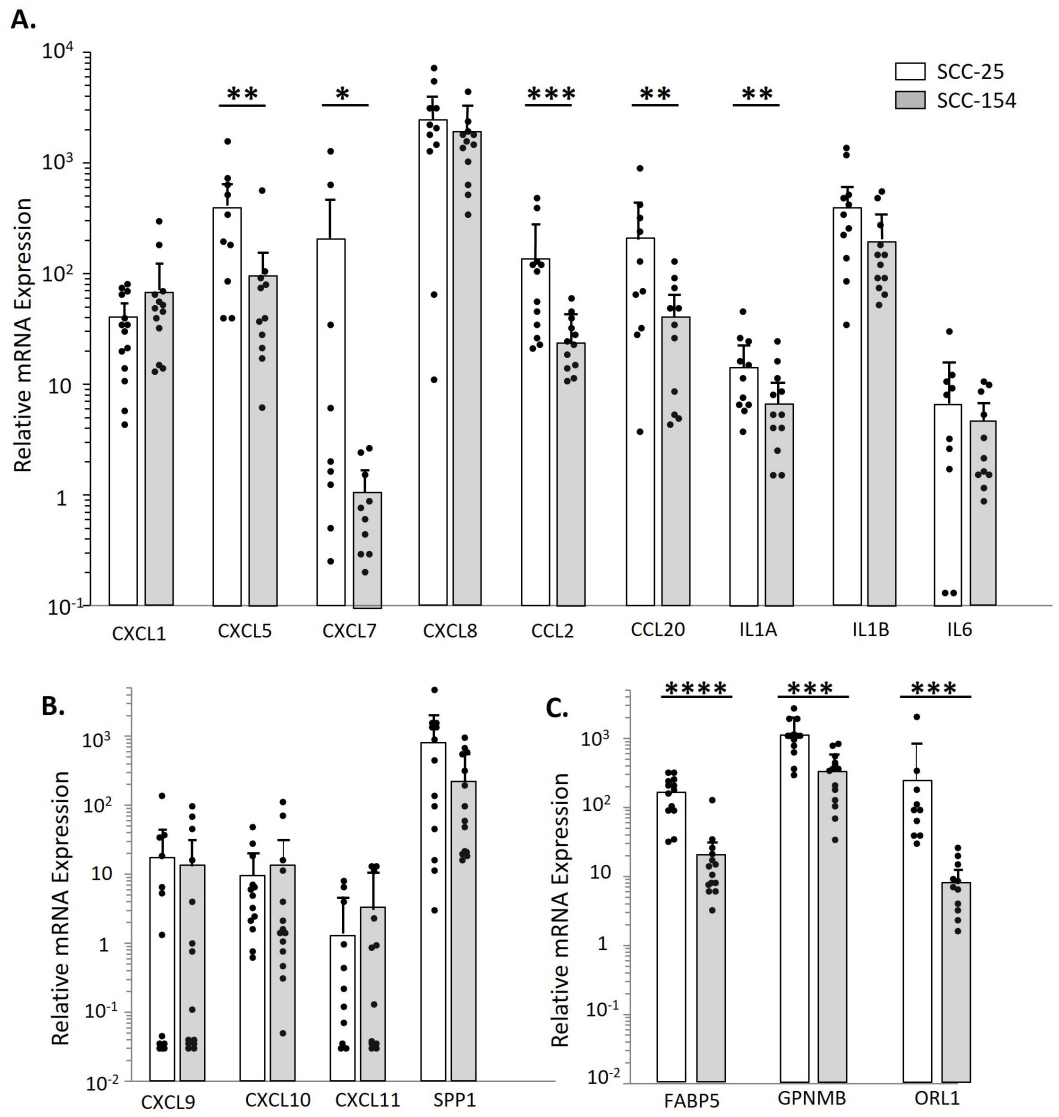
SCC-154 and SCC-25 derived TAMs express proinflammatory and pro-tumor markers observed during CM stimulation. To examine TAM cytokine/ chemokine expression levels, Monocytes were isolated from healthy control blood by negative selection. Monocytes were co-cultured with either SCC-25 or SCC-154 spheroids for 7 days at 10^4^ cells/spheroid. Spheroids were then dissociated with Accutase and stained with CD45, and flow sorted. Recovered CD45+ cells were pelleted, harvested for RNA and transcript levels were analyzed by qPCR. Expression levels are relative to GAPDH. **(A)** Expression of select cytokines and chemokines shown in **Figure 3**. **(B)** Relative expression of CXCL9/10/11 and SPP1. **(C)** Expression of metabolic markers that affect T-cell functions. Differential expression between spheroid types was assessed using a student’s T-test. *p<0.05, **p<0.01, ***p<0.001, ****p<0.0001.

We analyzed TAMs derived from controls for mRNA expression of cytokines, chemokines and other immune molecules that had been elevated by CM stimulation of monocytes in the scRNA-seq experiment or the expanded cohort studies (**Figure 6**). The cytokines and chemokines were expressed by the TAMs, but in contrast to short-term monocyte stimulation, the SCC-25 TAMs often had higher levels than SCC-154 TAMS. Interestingly, *CXCL7*, which was not induced in monocytes with SCC-154 CM stimulation, was also at very low levels in the SCC-154 TAMs. *CXCL9/10/11* levels were markedly lower than *SPP1*, consistent with the downregulation of these three chemokines by tumor cell-secreted factor(s) in short-term stimulation of control monocytes (see **Figure 4**). Finally, the TAMs expressed moderate to high levels of *FABP5, GPNMB* and *OLR1*. FABP5 and GPNMB are indicative of an abnormal fatty-acid metabolism while all three markers play a role in T-cell regulation and are often elevated in tumors (37–41).

### Expression of *CXCL9/10/11* in spheroid TAMs parallels OPC tumors. Resistance to down-regulation in tumors correlates with survival

**Figure 7:**
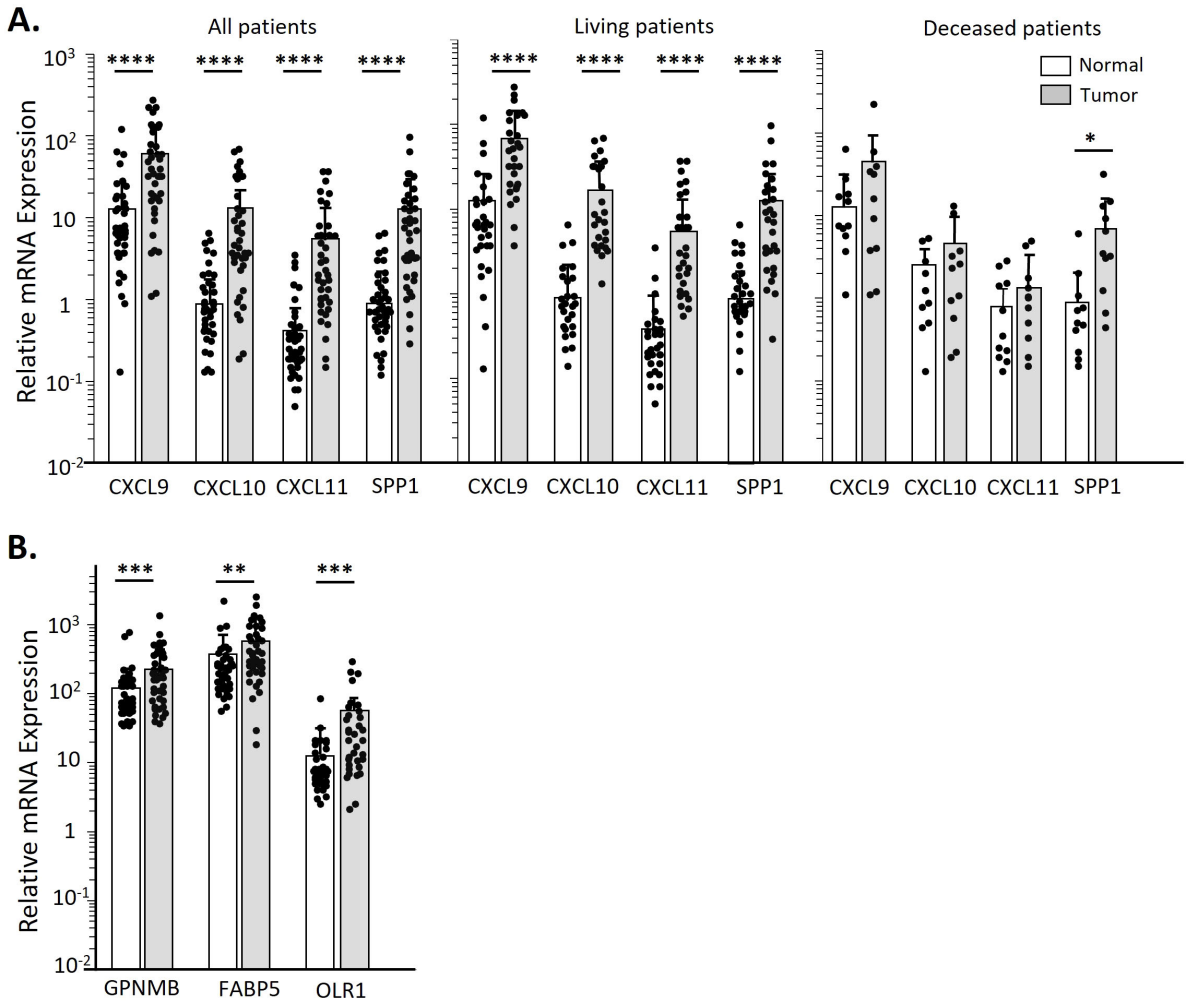
OPC biopsies have higher CXCL9/10/11 and SPP1 transcript levels over clinically normal tissue only in patients still living. Total RNA was isolated from matched sets of OPC tumor biopsies and normal tissues. Chemokine transcript levels were assessed by qPCR and expressed relative to GAPDH. **(A)** Transcript levels of CXCL9/10/11 and SPP1 in all patients in our OPC cohort by tissue type. Additional panels show data stratified by patient survival. **(B)** Expression levels of altered lipid metabolic genes in all OPC patients by tissue type. Data was analyzed by a Paired two-tailed T-test. *p<0.05; **p<0.01, ***p<0.001, ****p<0.0001.

We measured the expression of *CXCL9/10/11* in OPC tumors and clinically normal adjacent tissues, to confirm that our results with spheroids were consistent with *in vivo* expression. As seen in **Figures 7A, B** when considering either all patients or patients who did well with treatment, the tumors expressed significantly higher levels of these three chemokines than the adjacent tissues. However, this differential was much less in tumors from patients who subsequently died of their disease (**Figure 7A**), suggesting that tumors in those patients may have downregulated expression of the chemokines. We also measured expressions of *FABP5, GPNMB* and *OLR1* in the tumors and adjacent tissues, and found that they were expressed at significantly higher levels in the tumors than in the adjacent normal tissues, again confirming the ability of spheroids to mimic the *in vivo* tumor.

### Spheroid-induced TAMs suppress T-cell proliferation

**Figure 8:**
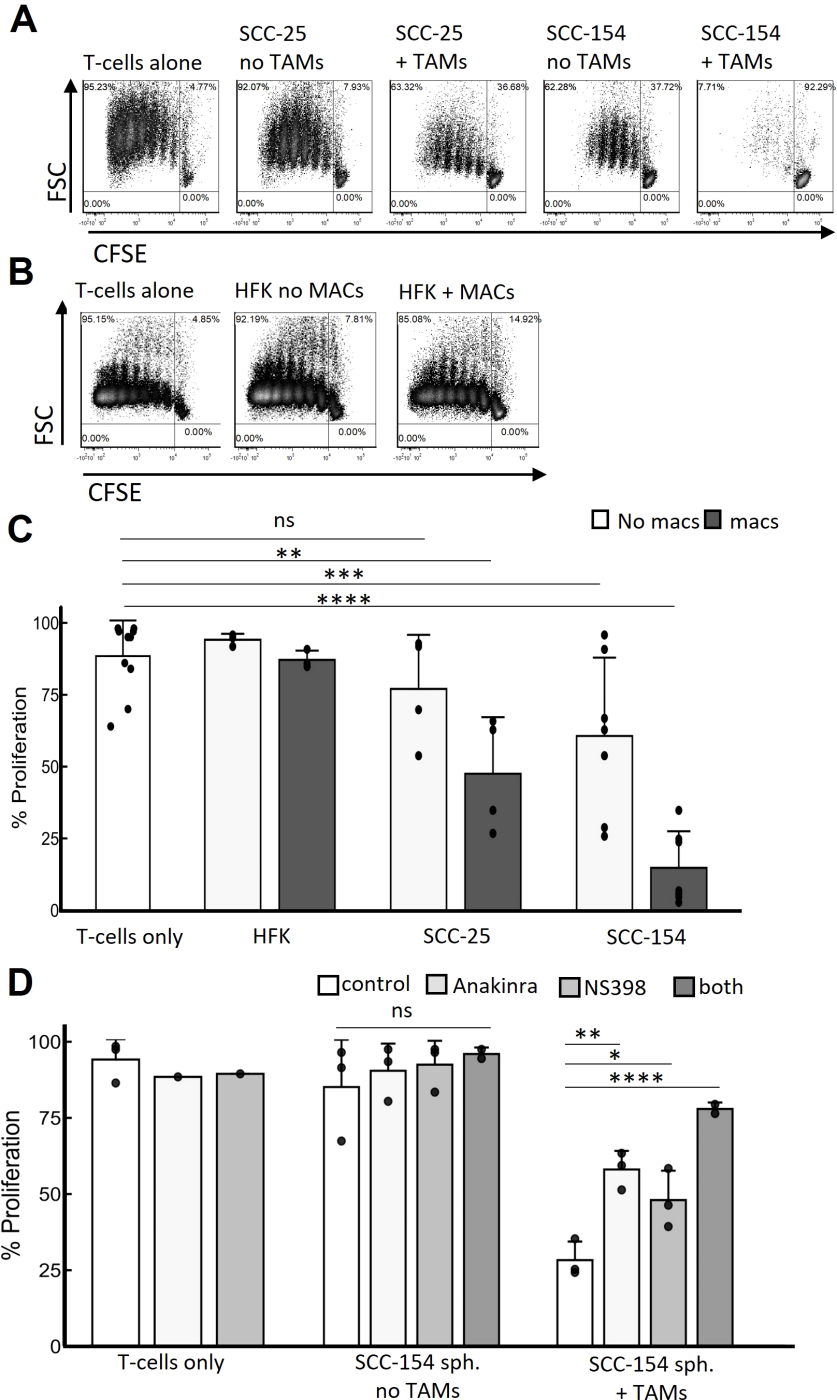
TAMs are major mediators of T-cell suppression that IL1α and COX-2 inhibition partially rescue. Spheroids were grown for 5 days in E-media +10% FCII. Control monocytes were isolated from PBMC by negative selection and co-cultured with spheroids for 7 days. Total naïve allogenic T-cells were isolated from PBMC also by negative selection, were stained with CSFE, activated with anti-CD3 beads and cocultured with the spheroids containing TAMs or not for an additional 6 days. Spheroids were then dissociated with Accutase and T-cell replication was assessed by CSFE signal decay. **(A)** Representative contour density plots of T-cell replication during coculture with SCC-25 or SCC-154 derived spheroids with or without TAMs. **(B)** Similar experiment but using human foreskin keratinocyte- derived spheroids in the presence and absence of control monocyte-derived macrophages. **(C)** Quantification of T-cell replication experiments from **(A, B)** Linear mixed effects model was used to test for significance. Significance was determined using a linear mixed effects model adjusting for multiple comparisons. **(D)** Quantification of T-cell replicative suppression in SCC-154 derived spheroids co-cultured in the presence or absence of TAMs using NS-398 (1μM) and Anakinra (25 ng/mL) to block PGE_2_ and IL-1α signaling, respectively. Significance was determined using an ANOVA with Tukey pairwise *post-hoc*. *p<0.05; **p<0.01, ***p<0.001, ****p<0.0001, ns, not significant.

Finally, we assessed the effect of monocyte-derived TAMs on T-cells. T-cell receptor crosslinking and CD28 ligation induced by anti-CD3 and anti-CD28 antibodies induces T-cell proliferation (42). Resting T-cells that had been activated with anti-CD3 and anti-CD28 antibodies and cultured with SCC-154 spheroids showed suppressed proliferation, indicating tumor driven immunosuppressive effects of these spheroids (**Figure 8**). Moreover, when these T-cells were exposed to spheroids that contained TAMs, the T-cells were more profoundly suppressed than when they were exposed to SCC-154 spheroids alone. In contrast, SCC-25 spheroids did not interfere with T-cell replication, and spheroids plus TAMs only partially inhibited proliferation (**Figure 8**). In contrast, keratinocyte spheroids containing macrophages induced from monocytes had no effect on T-cell proliferation. To begin to address mechanism, we added an IL-1 inhibitor, a COX-2 inhibitor, or the combination to SCC-154 spheroids at the same time as the monocytes. Either inhibitor was able to significantly reduce the inhibition of T-cell proliferation, and the combination was even more effective, suggesting that the functional differentiation phenotype of the TAMs had been altered (**Figure 8D**).

## Discussion

Monocyte activation and differentiation into macrophages is highly plastic. This plasticity is determined in large part by the extracellular environment, resulting in a varied repertoire of molecules the cells express on their membranes, and the immune mediators they secrete (43). As monocytes infiltrate solid tumors they are activated, educated by the tumor cells, and then differentiate into pro-tumor TAMs. These suppressive cells represent a major immunoregulatory barrier to effective tumor treatment and positive patient outcomes. While much has been learned regarding TAM diversity and functionality, the mechanisms regulating initial monocyte activation and TAM differentiation have not been adequately studied. Early monocyte education by tumors, and the monocytes that are ultimately released into the peripheral blood are impacted by the repertoire of tumor-derived mediators released into the blood and lymphatics. The process of tumor education of monocytes is less well characterized. In this study, we examined transcriptional responses of monocytes in an early education model where isolated monocytes were exposed to media conditioned by OPC cell lines, and we focused primarily on expression of select cytokines/chemokines. We identified TAM-related genes expressed early in CM-stimulated monocytes at the single cell level and expanded those findings with monocytes from a cohort of OPC patients, confirming the CM-driven differential expression observed during scRNA-seq. Shifting to a TAM differentiation model by co-culture of monocytes with OPC cell line-derived spheroids allowed us to describe monocyte phenotypes and cytokine/chemokine expression pattern likely to shape the TME during early tumor establishment. Finally, we showed that those TAMs are capable of suppressing T-cell proliferation within spheroids.

The impact of factors secreted by tumors on monocytes begins early in oncogenesis (44) and continues as the tumor grows. Alteration of steady state monocytes to activated monocyte subgroups occurs early on during tumor development. Tumors induce monocyte alteration in the bone marrow, spleen, and in the peripheral blood (44). These activated monocytes are likely being “primed” by tumors to become tumor-supporting TAMs. This shift from the normal monocyte phenotype is illustrated by the significant reduction of “classical” monocytes and the increase in “non-classical” monocytes in patients with OPC compared to controls, shown in **Figure 1**. This shift is also apparent in the scRNA-seq results in **Figure 2**. Monocyte and TAM diversity was recently revised based on extensive transcriptomics approaches, leading to description of novel subsets (31, 45). Those studies confirmed the CD14/CD16 expression paradigm for “classical” and “non-classical” monocytes while at the same time revealing subtler diversity between those cells and the less well understood “intermediate” monocytes (31).

After incubation with tumor- cell conditioned media or with IL-1α, the monocytes from both a patient and a control in our study were transcriptionally most like Villani et al. (31) groups Mono1 (classical) and Mono2 (non-classical) cells with trace numbers of Mono3 and dendritic cell subgroups. However, within these large categories were smaller clusters of cells, based on their transcriptional patterns (**Figure 2A**), that reflected the varied stimuli they were exposed to (**Figure 2B**). The cluster 6 monocytes were defined by high expression of multiple pro-tumor genes (*IL1B, SERPINB2, CCL2, CXCL1, CXCL5, CXCL8, CCL3, CCL20*) (**Figure 2C**). This cluster was biased 3:1 with monocytes from the patient, and as seen in the violin plots (**Figure 2D**), expression of many of these cytokines/chemokines was much higher in the patient than the control. This could be due to prior “priming” of a subset of the patient’s monocytes by the tumor *in vivo.* Interestingly, within our single cell data set we noticed patterns of markers that indicate a division of tumor-supportive mechanisms across monocyte clusters. Inflammatory cytokines/chemokines were primarily expressed by Cluster 6 monocytes that may be responsible for coordinating recruitment of other detrimental cell populations. Clusters 0 and 1 cells did not express this group of markers. Instead, the highest expressing transcripts were pro-fibrotic, ECM-related markers including FN1, COL23A1 and TGFBI after SCC-154 or IL-1α treatments. Even after short-term CM exposure, we start to see a diversity of monocytes that may influence TAM function in OPC.

The “priming” or shift in transcriptional pattern to a more pro-tumor phenotype by patient monocytes *in vivo*, was supported by CM stimulation of a much larger cohort of patients’ and control monocytes, as shown in **Figure 3**. Overall patterns of expression induced by the CM were similar for control and patient monocytes, but expression levels of several of the cytokines/chemokines was significantly higher in patient monocytes prior to stimulation (*CXCL5, CXCL8, CCL2*, and *IL1A* and *IL1B*), and were also more robustly expressed by patient monocytes following stimulation (*CXCL1, CXCL5, CXCL8* and *CCL2*). These chemokines play multiple roles in the TME. In addition to influencing immunocyte populations, they can influence behavior of the tumor cells themselves (enhancing cell migration and invasion, promoting cell survival, and promoting stem-cell properties), further elevating their importance. CCL2 is a key monocytic chemotactic factor, influencing monocyte mobility and activation and can help recruit MDSCs (46–48). CXCL1, CXCL5 and CXCL8 can recruit neutrophils and MDSCs and contribute to immune suppression and can also promote tumor cell migration and invasion of some tumor types (18, 49–51). Of interest, CXCL1 has been shown to be expressed and secreted from precancerous fibrous lesions in the oral submucosa (52) in response to inflammatory cytokines including IL-1α, and we had previously reported that IL-1α is present in the conditioned medium of both SCC-25 and SCC-154 (26). We suggest that CM-stimulated monocytes are a model for understanding activated, pre-TAM monocytes *in vivo* that could start calling in pro-tumor PMNs and MDSCs as soon as they enter the tumor and before they become bone fide TAMs.

CXCL9, -10, -11 are CXCR3 ligands, expressed by several different cells within a tumor, that recruit T-cells and NK cells, with CXCL9 and 10 primarily recruiting activated CD4^+^ and CD8^+^ effector cells and CXCL11 recruiting or promoting development of T-regs (53, 54). Low levels of these chemokines within in a tumor contributes to a “cold” tumor landscape unable to respond to immune therapies such as checkpoint inhibitors, while high CXCL9/10 contributes to “hot” tumors where effector cells are present but may not function properly. Low levels of CXCL9 or -10 have been correlated with poor outcomes in many types of tumors (55, 56).

SPP1 plays multiple roles within a tumor, depending on the cell type, including enhancing M2-like macrophage infiltration (57). A recent study reported that *CXCL9* and *SPP1* are very rarely expressed in the same monocyte, and that the balance of *CXCL9^+^* vs *SPP1^+^* TAMs predict patient outcome for squamous cell carcinomas of the head and neck (33). Our studies with OPC patients’ tumors (**Figure 7**) found that in those patients who died of their disease the levels of *CXCL9/10/11* expression in tumors were low, comparable to adjacent normal tissues, while levels of SPP1 were significantly higher in the tumors than the adjacent tissue. *CXCL9/10/11* are expressed in response to interferon-γ (IFN-γ) while regulation of *SPP1* expression appears to vary depending on cell type (33), but the rare expression of both *CXCL9/10/11* and *SPP1* in the same monocyte suggests more complex and possibly mutually exclusive gene expression programs are present in these cells.

We also described the novel observations (**Figure 4**) of a distinct group of patients (Group 1) whose blood monocytes express very high levels of *CXCL9/10/11* that are resistant to downregulation by SCC25- or SCC154-CM, unlike most OPC patients and control monocytes. This pattern could reflect monocyte resistance to tumor-education but mechanisms for this remain unknown. Despite this, considering known functions of CXCL9 and CXCL10, maintenance of high levels of these chemokines in tumor cells or TAMs lead to a greater abundance of effector T-cells associated with diseased tissues. Expression of these cytokines contribute in part to the immunological landscape of a patient’s tumor, and those with T-cells present respond better to checkpoint inhibition therapeutics and show better outcomes. Additional time will be needed to determine whether Group 1 patients have better outcomes. Future studies that define the mechanism of the downregulation, and the ability of some patients’ monocytes to resist it, could be used to develop targeted therapies to improve outcome.

Initial priming or programming of monocytes *in vivo* by tumor-derived factors may help determine the phenotype of TAMs in a tumor. Studies using single-cell transcriptomics to examine TAM diversity found two major subtypes (defined by either *C1Q* or *SPP1* expression) and two minor subtypes (defined by either *CCL18* or *FCN1* expression) (45). While no cluster in our analysis of monocytes stimulated with tumor-cell CM (**Figure 2**) was defined by those four markers, we observed increases in some of the genes, including a modest upregulation of *SPP1* in cluster 7 and a marked increase in *FCN1* in cluster 0 cells. Moreover, *APOE, MRC1* and *CD68*, all genes expressed in the *C1Q+* TAM subset, increased with CM stimulation but *C1Q* itself did not. The pattern of cytokines/chemokines induced by SCC-25 and SCC-154 CM were also expressed in TAMs generated in SCC-25 and SCC-154 spheroids. Moreover, *GPNMB, FABP5* and *OLR1*, three other genes that play important roles in the TME of some tumors (58–60) and that were upregulated in OPC tumors compared to adjacent tissues (**Figure 7**), were expressed in response to CM in our sc-RNAseq study (**Figure 2**) and were highly expressed in the TAMs isolated from the spheroids (**Figure 5**). Additionally, many markers we observed that defined our monocyte clusters (*FABP5, FN1, IL1RN, CCL3, CCL4, CXCL8, CXCL3, IL1B*) during transient, short-term CM stimulation, were elevated in either PD-L1^pos^ or PD-L1^neg^ TAMs isolated and sequenced from breast cancer (61). Thus, we conclude that exposure of monocytes to soluble factors secreted from tumors, even before the monocytes enter the tumor, could initiate TAM differentiation.

Finally, we have shown that TAM function in spheroids varies, dependent on factors secreted by the tumor cells. TAMs differentiated by SCC-154 spheroids were much more efficient in suppressing T-cell proliferation than TAMs differentiated by SCC-25, while macrophages that differentiate within HFK spheroids had no suppressive effect on T-cells (**Figure 8**). Treating the monocytes in the SCC-154 spheroids with either an IL-1 or a COX-2 inhibitor while they were differentiating into TAMs partially blocked their ability to suppress T-cell proliferation and the combination of these inhibitors was more effective than either one alone. While the influence of PGE_2_ (a downstream product of COX-2 activity) on macrophage M1-like polarization remains unclear, it does facilitate M2-like polarization (62–64). We previously showed that SCC-154 secreted significantly higher levels of IL-1α than SCC-25, and that IL-1α induced expression of COX-2 in monocytes (26). The role of IL-1α in tumor biology is complex, but many studies suggest it has pro-tumor activity (65). Additionally, we acknowledge that other TAM-mediated, tumor-supportive mechanisms are most likely contributing to T-cell dysfunction, beyond IL-1α and PGE_2_ activity. Induction of LILRB4 transcript in a subset of our CM-stimulated monocytes could represent an additional IL-1α/PGE_2_ independent T-cell suppressive mechanism (66–68). Future studies will be needed to determine the molecular mechanisms involved in TAM suppression of T-cells in the spheroid model. We are currently developing a mouse OPC line overexpressing IL-1α, to determine its role in the TME *in vitro* and *in vivo*.

Although PGE_2_ and IL-1α signaling do not account for the entirety of T-cell suppression in our OPC spheroid model, we believe these are druggable targets to help block local immunosuppression. Safe, effective, FDA approved small molecule compounds and biologics exist to inhibit PGE_2_ synthesis (Celebrex, a potent and specific COX-2 inhibitor) or IL-1α signaling (Rilonacept and Anakinra, inhibitors of the IL-1α protein and IL-1R antagonist respectively) are routinely used for other pathologies. Repurposing and coupling such drugs with standardized therapies may boost favorable outcome.

Spheroid models do not capture the full complexity of the TME. They do represent a valid, more physiologically accurate cell-cell relationship over study of conventional 2-dimentional monolayers (69, 70). In conclusion, we have studied the progression from naïve monocytes to activated monocytes secreting cytokines and chemokines that promote tumor growth, to TAMs that can suppress T-cells *in vitro*, mediated by factors secreted from OPC tumor cell lines. Future studies, beyond the scope of this study, will be needed to determine the mediators secreted by the tumor cells and the molecular processes involved at each step in this process.

## Data Availability

The datasets presented in this study can be found in online repositories. The names of the repository/repositories and accession number(s) can be found below: https://www.ncbi.nlm.nih.gov/, GSE281098.
