# Supplementary material for: Oropharyngeal carcinomas induce circulating monocytes to express a TAM-like pro-tumor expression profile that suppresses T-cell proliferation
Source: Front Immunol. 2025 Mar 19;16:1539780. doi: 10.3389/fimmu.2025.1539780 (PMC11961958; doi:10.3389/fimmu.2025.1539780)
Supplement: Supplementary file 1 [file Image1.pdf]

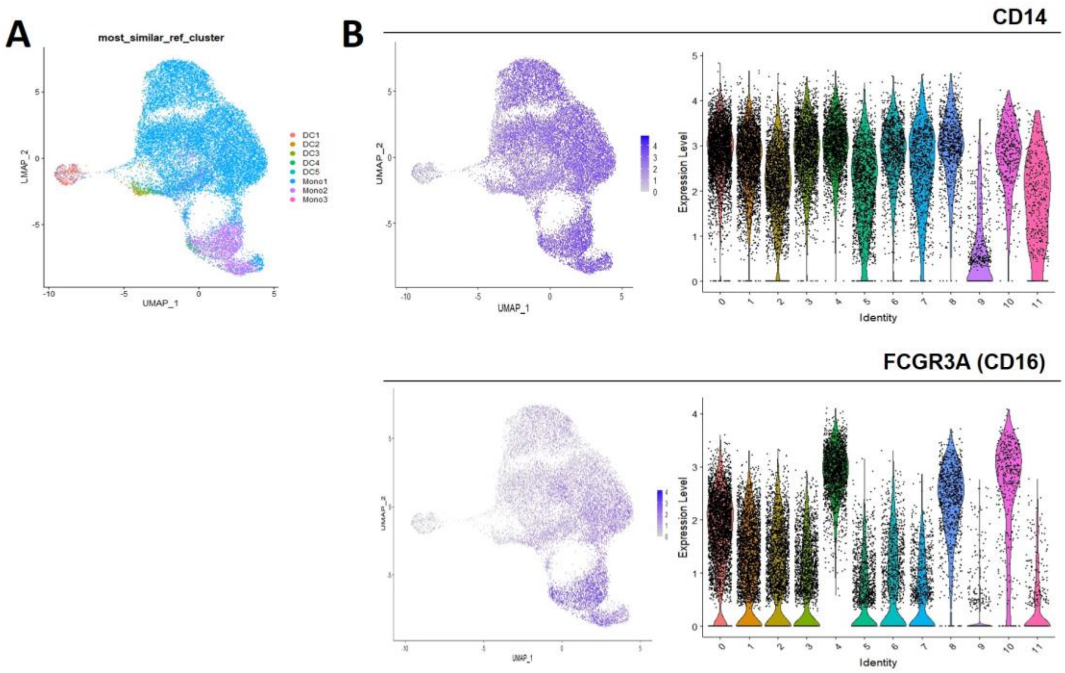

Supplemental Figure 1. **Monocytes observed in scRNA-sequencing experiments can be subdivided into major ‘Classical’ (Mono1/CD14+) and minor ‘Non-classical’ (Mono2/CD14+/CD16+) with trace numbers of Mono3 and DC subsets.** A) UMAP showing each cell from our scRNA-seq data set assigning each cell to a monocyte or DC subtype described in Villani et al., 2017 which agrees with ‘Classical’, ‘Non-classical’ subtype paradigm based on B) CD14/CD16 expression.
